# Determination of the cutoff point for Smartphone Application-Based Addiction Scale for adolescents: a latent profile analysis

**DOI:** 10.1186/s12888-023-05170-4

**Published:** 2023-09-16

**Authors:** Pu Peng, Zhangming Chen, Silan Ren, Yi Liu, Ruini He, Yudiao Liang, Youguo Tan, Jinsong Tang, Xiaogang Chen, Yanhui Liao

**Affiliations:** 1 Department of Psychiatry, Sir Run Run Shaw Hospital, Zhejiang University School of Medicine, 3 East Qingchun Road, Hangzhou, 310016 Zhejiang China; 2 Department of Psychiatry, National Clinical Research Center for Mental Disorders, and National Center for Mental Disorders, The Second Xiangya Hospital of Central South University, Changsha, 410011 Hunan China; 3 Department of Nursing, Sichuan Vocational College of Health and Rehabilitation, Zigong, Sichuan China; 4 Department of Psychiatry, Zigong Mental Health Center, Zigong, Sichuan China

**Keywords:** Smartphone Application-Based Addiction Scale, Problematic smartphone use, Problematic social media use, Internet gaming disorder

## Abstract

**Backgrounds:**

The Smartphone Application-Based Addiction Scale (SABAS) is a validated 6-item measurement tool for assessing problematic smartphone use (PSU). However, the absence of established cutoff points for SABAS hinders its utilities. This study aimed to determine the optimal cutoff point for SABAS through latent profile analysis (LPA) and receiver operating characteristic curve (ROC) analyses among 63, 205. Chinese adolescents. Additionally, the study explored whether PSU screening with SABAS could effectively capture problematic social media use (PSMU) and internet gaming disorder (IGD).

**Method:**

We recruited 63,205. adolescents using cluster sampling. Validated questionnaires were used to assess PSMU, IGD, and mental health (depression, anxiety, sleep disturbances, well-being, resilience, and externalizing and internalizing problems).

**Results:**

LPA identified a 3-class model for PSU, including low-risk users (38.6%, *n* = 24,388.), middle-risk users (42.5%, *n* = 26,885.), and high-risk users (18.9%, *n* = 11,932.). High-risk users were regarded as “PSU cases” in ROC analysis, which demonstrated an optimal cut-off point of 23 (sensitivity: 98.1%, specificity: 96.8%). According to the cutoff point, 21.1% (*n* = 13,317.) were identified as PSU. PSU adolescents displayed higher PSMU, IGD, and worse mental health. PSU screening effectively captured IGD (sensitivity: 86.8%, specificity: 84.5%) and PSMU (sensitivity: 84.5%, specificity: 80.2%).

**Conclusion:**

A potential ideal threshold for utilizing SABAS to identify PSU could be 23 (out of 36). Employing SABAS as a screening tool for PSU holds the potential to reliably pinpoint both IGD and PSMU.

**Supplementary Information:**

The online version contains supplementary material available at 10.1186/s12888-023-05170-4.

## Introduction

The smartphone has become the main gadget for assessing the internet. 86.11% of the world’s population owns a smartphone [1]. In China, 97.5% of internet users prefer using smartphones to access the internet [2].

While smartphones offer convenience, increasing studies indicate that problematic smartphone use (PSU) could lead to various dysfunctional manifestations, particularly among adolescents [3]. While the recognition of PSU as an addiction remains a contentious issue [4], there is broad consensus that PSU represents an individual's inability to regulate their smartphone usage, subsequently leading to adverse consequences in their daily life [5]. PSU represents the most prevalent form of problematic technology use (PTU) and affects approximately 21% of adolescents worldwide [6]. PSU is closely associated with various psychological outcomes in adolescents, such as depression [7], anxiety [8], sleep disturbance [9], impaired mental wellbeing [10], low resilience [11], and externalizing problems [12], which warrants early identification and timely intervention for PSU.

Reliable and validated measurement tools are essential in identifying and preventing PSU [5]. One frequently used and well-established tool for PSU assessment is the Smartphone Application Based Addiction Scale (SABAS) [13]. This brief questionnaire is designed based on the addiction components proposed by Griffith and encompasses six items assessing salience, tolerance, mood modification, relapse, conflict, and withdrawal symptoms of PSU [13]. SABAS has demonstrated excellent psychometric properties across different cultures [13–19]. Compared to other commonly used measures of PSU, such as the Smartphone Addiction Scale (26 items) [20], Smartphone Addiction Scale-Short version (10 items) [21], and Smartphone Addiction Inventory (26 items) [22], SABAS stands out as the most concise scale. Its brevity makes it particularly suitable for screening PSU in epidemiological studies.

However, the lack of established cutoff points for SABAS to detect PSU hinders its utilities. With a clear cutoff point, SABAS could effectively distinguish PSU from normal users, which facilitates future epidemiological research (e.g., estimation of the PSU prevalence) and intervention (e.g., determining the PSU cases and providing interventions). Previous studies in Bangladeshi samples have employed an empirical cutoff point of 21 for SABAS to detect PSU [23, 24]. However, the optimal cutoff point for SABAS has not been definitively established.

Hence, the first objective of our study was to determine the cutoff point for SABAS among a large sex-balanced sample of Chinese adolescents. Latent profile analysis (LPA) was utilized, which was an epidemiological approach to determine the cutoff point when a clinical interview is not available [25]. It allows for the identification of distinct groups of smartphone users based on their responses to SABAS, with the group exhibiting the highest levels of PSU being identified as the "PSU" cases for determining the optimal cutoff point. To date, numerous studies have applied the LPA approach to derive the optimal cutoff point for measurement tools, such as Bergen Social Media Addiction Scale (BSAMS) [26], online social networking addiction scale [27], Dimensional Anhedonia Rating Scale [28], and Center for Epidemiologic Studies Depression Scale [29].

Our second objective was to determine the ability of PSU screening with SABAS to capture problematic social media use (PSMU) and internet gaming disorder (IGD). PSMU and IGD are common PTUs related to specific technology in adolescents [6, 30]. Similar to PSU, PSMU and IGD are closely associated with mental distress [31–33]. Various assessment tools for IGD and PSMU have been developed. However, utilizing multiple questionnaires may increase the burden of the screening. As per the cognitive-behavioral model proposed by R.A. Davis [34], PTU can be categorized into generalized conditions, such as internet addiction and PSU, and specific conditions that involve the excessive use of particular internet functions, such as IGD, PSMU, and online gambling. Recent research suggests that PSU could act as an umbrella construct encompassing more specific types of PTU, including PSMU and IGD [35]. Given the ubiquitous use of smartphones as the principal conduit for internet access among adolescents, it is plausible to consider PSU as an early stage of PTU. Adolescents presenting with specific PTUs such as IGD and PSMU are likely to also meet the criteria for PSU. One recent study in American samples also suggested that screening for problematic internet use, a broad, generalized condition, could identify PSMU and IGD with high sensitivity [36]. However, limited studies shed light on the overlap between PSMU, PSU, and IGD in Chinese adolescents. If it is true that detecting PSU with SABAS could also identify those with IGD and PSMU, SABAS could be used as an initial screening tool, followed by targeted assessments focusing on specific PTU.

While the study is predominantly explorative, we have three main hypotheses: (1) LPA will successfully identify a subgroup characterized by higher SABAS scores (PSU cases) than other subgroups; (2) Compared to non-PSU cases, PSU cases will exhibit much worse mental health; and (3) There will be a substantial overlap between PSU, IGD, and PSMU cases. Most IGD and PSMU cases will also meet the criteria for PSU.

## Method

### Study procedure and participants

This school-based study was conducted from September to December 2020 in Zigong, a city located in the south of Sichuan, China. Zigong includes four districts and two counties. We engaged adolescents from two districts (Gongjing and Ziliujing) and one county (Fushun). Using a cluster sampling approach, students from all junior and senior high schools within these selected areas were recruited for the study. To ensure the validity of the surveys, investigators and head teachers of the school received training on the survey process and questionnaire. They were responsible for introducing the study purpose, answering students’ questions when necessary, and observing the survey process. The questionnaire was electronic and participants completed the survey in the computer room of selected schools.

Informed consent was obtained from all participants and their parents (for students who were younger than 18) prior to their involvement in the study. All participants, including their parents, were reassured of their right to decline participation or to discontinue their involvement at any point. They were thoroughly briefed about the study’s objectives, procedures, measurements, potential risks, and benefits before the commencement of the survey. The protocol was approved by the Ethics Committee of Zigong Mental Health Center [No. 2020–8-01].

### Measurements

The following basic information was collected through self-designed questionnaires: age, gender, school type (junior high/senior high), residence (urban/rural), only child (yes/no), left-behind children (yes/no), current drinking status, and current smoking status.

We used SABAS to evaluate PSU. SABAS consisted of six items, measuring salience (the extent to which the smartphone becomes the most important thing), tolerance (increased time spent on the smartphone), mood modification (using the smartphone to cope with mood problems), relapse (repeated failure to reduce smartphone use), withdrawal symptoms (feeling upset, irritable, and angry when unable to use the smartphone), and conflict (conflicts with others due to smartphone use) [13]. SABAS applied a 6-point Likert scale, ranging from 1 (strongly disagree) to 6 (strongly agree). The total scores of SABAS ranged from 6 to 36, with higher scores suggesting more PSU risk. The Cronbach’s α of SABAS was 0.876.

We used BSMAS and the Internet Gaming Disorder Scale-Short Form (IGDS9-SF) to measure PSMU and IGD, respectively. These scales have strong validity and are widely used in the Chinese population [15, 37]. BSMAS consists of six items measuring components of addiction. IGDS9-SF consists of 9 items based on the DSM-5 criteria for IGD. Higher scores on these scales indicate more severe problematic use. Following previous studies [26, 38], we used cutoff points of 24 for BSMAS and 32 for IGDS9-SF to determine the presence of PSMU and IGD. The Cronbach’s α for IGDS9-SF and BSMAS were 0.913 and 0.867 respectively, indicating high internal consistency.

Mental well-being and psychological distress were measured via the following validated questionnaires: 9-item Patient Health Questionnaire (PHQ-9) for depression, 7-item Generalized Anxiety Disorder Scale (GAD-7) for anxiety, Pittsburgh sleep quality index (PSQI) for sleep disturbances, self-reported version of Strengths and Difficulties Questionnaires (SDQ) for internalizing and externalizing problems (emotional problems, conduct problems, peer problems, hyperactivity, and prosocial behavior), Connor-Davidson Resilience Scale 10 (CD-RISC-10) for resilience, and Warwick-Edinburgh Mental Well-being Scale (WEMWBS) for mental wellbeing. Previous studies have confirmed their satisfactory psychometric properties in Chinese populations [39–42]. Following previous studies [43, 44], a cutoff point of 10 for PHQ-9, 10 for GAD-7, and 6 for PSQI was used to determine the presence of depression, anxiety, and sleep disturbances.

### Statistical analysis

First, we used latent profile analysis (LPA) to identify groups of participants (latent classes) with similar levels of risk of PSU based on their responses to the SABAS items. We estimated models with 2–6 latent profiles using the R package “tidyLPA”. Following LPA guidelines and previous studies [26, 45], the following indicators were considered when deciding the optimal model: (1) Lower Akaike Information Criteria (AIC) and Bayesian Information Criteria (BIC); Scree plot was used to visualize the change. (2) Entropy > 0.8; and (3) The bootstrap likelihood ratio (BLRT). BLRT *p*-value < 0.05 suggested a significant improvement in model fit compared to the solution with one fewer class. We assessed the validity of the LPA identification of probable PSU cases (the latent class with the highest risk) by comparing external criteria (IGD and PSMU), impaired mental health (resilience and mental wellbeing), and psychological distress (depression, anxiety, sleep problems, and other internalizing and externalizing problems) among the LPA classes.

Second, following the previous study [27], we performed a sensitivity analysis to determine the optimal cut-off point of the SABAS using ROC curves. In this analysis, the probable PSU cases (the subgroup with the highest SABAS scores) versus non-cases (all other subgroups) identified by LPA were utilized as the reference standard. Measures such as sensitivity, specificity, Youden’s index, accuracy, positive predictive value (PPV), and negative predictive value (NPV) were calculated for potential cutoff points of the SABAS scale based on this reference standard. The cutoff point yielding the highest Youden’s index was selected as the most optimal, which struck an appropriate balance between sensitivity and specificity in distinguishing probable PSU cases from non-cases. We then divided the participants into the positive (probable PSU cases) and negative (probable non-cases) groups based on the derived cut-off point of SABAS. We compared the basic information, problematic internet use, and mental health between the two groups. Cohen's d was calculated to determine the effect size for continuous data (0.2–0.5: small; 0.5–0.8: medium; > 0.8: large). For categorical data, crude odds ratio and 95% CI were calculated.

Finally, we assessed the overlap in positive cases of PSU, IGD, and PSMU and visualized the overlap with Venn Plot. We calculated the sensitivity and specificity of using the derived cut-off point for SABAS to detect IGD and PSU.

All statistical analysis was conducted on R software. The inter-group differences between LPA classes and positive/negative PSU participants were determined through chi-square tests, student t-tests, and ANOVA tests as appropriate. Tests were two-tailed, with *p* < 0.05 suggesting statistical significance.

## Result

### Sample characteristics

We recruited 63,487. students from 76 senior and junior high schools. After removing responses with missing data, 63, 205. participants who provided validated responses were included in the final analysis (Table 1). The sample was sex-balanced (girl: 32, 010; boy: 31, 195.), with a mean age of 14.3 years old. The majority of the participants were junior high school students (68%, *n* = 43, 373.) and lived in the city (67%, *n* = 42, 059.). Of the participants, 22% (*n* = 13,904.) were only children and 35% (*n* = 22,202.) were left-behind children. The prevalence of depression, anxiety, sleep disturbance, IGD, and PSMU was 23% (*n* = 14, 550.), 14% (*n* = 8813), 30% (*n* = 18, 647.), 2.87% (*n* = 1813), and 2% (*n* = 1290), respectively.

**Table 1:** Sample characteristics and latent profile analysis of the adolescents

**Variable**	**Overall**, *N* = 63,205^a^	**High risk users**, *N* = 11,932^a^	**Middle risk users**, *N* = 26,885^a^	**Low risk users**, *N* = 24,388^a^	***p*-value**^b^
**Gender, female**	32,007 (51%)	6,030 (51%)	13,724 (51%)	12,253 (50%)	0.2
**Age**	14.33 (1.65)	14.44 (1.54)	14.50 (1.65)	14.09 (1.67)	< 0.001
**School type**					< 0.001
Junior high school	19,832 (31%)	3,972 (33%)	9,665 (36%)	6,195 (25%)	
Senior high school	43,373 (69%)	7,960 (67%)	17,220 (64%)	18,193 (75%)	
**Current smoker, yes**	886 (1.4%)	360 (3.0%)	341 (1.3%)	185 (0.8%)	< 0.001
**Current drinker, yes**	6,718 (11%)	2,434 (20%)	2,917 (11%)	1,367 (5.6%)	< 0.001
**Living in urban, yes**	46,871 (74%)	8,923 (75%)	19,897 (74%)	18,051 (74%)	0.2
**Only children, yes**	13,904 (22%)	2,806 (24%)	5,831 (22%)	5,267 (22%)	< 0.001
**Left-behind children, yes**	22,202 (35%)	4,231 (35%)	9,591 (36%)	8,380 (34%)	0.006
**SDQ-Emotional Symptoms**	3.06 (2.49)	4.65 (2.61)	3.24 (2.33)	2.08 (2.12)	< 0.001
**SDQ-Conduct Problems**	2.24 (1.56)	3.08 (1.71)	2.27 (1.47)	1.80 (1.40)	< 0.001
**SDQ-Hyperactivity Inattention**	3.73 (2.21)	5.26 (2.13)	3.95 (1.99)	2.74 (1.97)	< 0.001
**SDQ-Peer problems**	3.16 (1.58)	3.42 (1.71)	3.15 (1.57)	3.05 (1.52)	< 0.001
**SDQ-Prosocial behavior**	7.24 (2.11)	6.55 (2.13)	7.10 (1.99)	7.73 (2.11)	< 0.001
**SDQ total Difficulties**	12.2 (5.8)	16.4 (5.7)	12.6 (5.1)	9.7 (5.0)	< 0.001
**PHQ9**	6.4 (5.2)	10.6 (5.9)	6.9 (4.6)	3.9 (4.0)	< 0.001
**WEMWBS**	46 (13)	40 (12)	45 (11)	51 (13)	< 0.001
**IGDS9SF**	15.3 (6.7)	22.0 (8.4)	15.6 (5.3)	11.7 (3.8)	< 0.001
**BSMAS**	11.0 (4.8)	15.3 (5.9)	11.5 (4.1)	8.4 (3.0)	< 0.001
**SABAS**	17 (7)	27 (3)	19 (3)	10 (3)	< 0.001
**CDRISC**	23 (9)	18 (8)	22 (8)	26 (10)	< 0.001
**GAD-7**	5.3 (4.3)	8.5 (4.8)	5.7 (3.8)	3.3 (3.4)	< 0.001
**PSQI**	4.2 (3.2)	6.4 (3.4)	4.6 (2.9)	2.8 (2.6)	< 0.001
**IGD, yes (IGDS9-SF ≥ 32)**	1,813 (2.9%)	1,558 (13%)	186 (0.7%)	69 (0.3%)	< 0.001
**PSMU, yes (BSAMS ≥ 24)**	1,274 (2.0%)	1,055 (8.8%)	166 (0.6%)	53 (0.2%)	< 0.001
**Sleep disturbance, yes (PSQI ≥ 6)**	18,647 (30%)	6,570 (57%)	8,662 (33%)	3,415 (14%)	< 0.001
**Depression, yes (PHQ-9 ≥ 10)**	14,550 (23%)	6,100 (51%)	6,347 (24%)	2,103 (8.6%)	< 0.001
**Anxiety, yes (GAD-7 ≥ 10)**	8,813 (14%)	4,158 (35%)	3,478 (13%)	1,177 (4.8%)	< 0.001

*SDQ* Strength and Difficulties questionnaire, *PHQ9* 9-item Patient health questionnaire, *WEMWBS* Warwick-Edinburgh Mental Well-being Scale, *IGDS9-SF* 9-item Internet Gaming Disorder scale Short Form, *BSMAS* Bergen Social Media Addiction Scale, *SABAS* Smartphone-Application Based Addiction Scale, *GAD-7* 7-item Generalized Anxiety Disorder Scale (GAD-7) for anxiety, *PSQI* Pittsburgh sleep quality index, *CD-RISC* Connor-Davidson Resilience Scale, *PSU* problematic smartphone use, *IGD* internet gaming disorder, *PSMU* problematic social media use

^a^n (%); Mean (SD)

^b^Pearson’s Chi-squared test; ANOVA tests

### Latent profile analysis

**Fig. 1:**
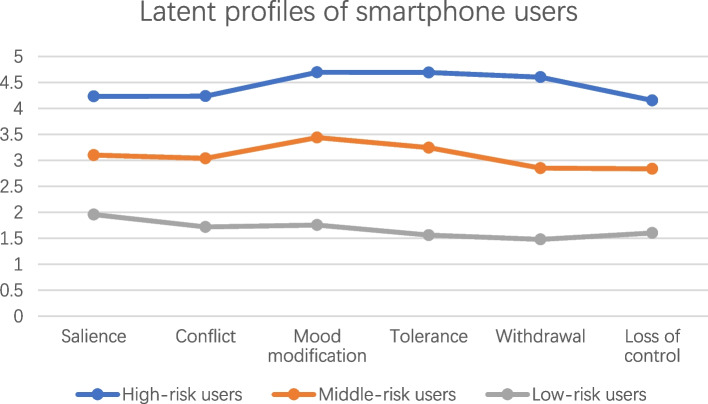
The latent profile of PSU based on SABAS scores The X-axis represented the items of SABAS. The Y-axis represented the scores on each item of SABAS

We evaluated LPA models ranging from two to six classes to determine the optimal number of classes. Table 2 presents the AIC, BIC, SA-BIC, entropy, and results of the BLRT for each model. Although AIC, BIC, and SA-BIC progressively decreased with the addition of profiles, the scree plot (Figure S1) indicated the 3-class and 4-class models as potential inflection points. However, the 4-class, 5-class, and 6-class models exhibited less desirable entropy (below 0.8), leading us to select the 3-class model. The entropy of the 3-class model was 0.82, indicating a robust classification accuracy. Figure 1 depicts the three-class model of PSU. The first class (“low-risk users”) included 24,388. (38.6%) participants who reported “disagree” or “strongly disagree” on all SABAS items. The second class comprised 42.5% of the sample (*n* = 26,885.) who endorsed “slightly disagree” on the six items and were thus named “middle-risk users”. The third class consisted of 11,932. adolescents (18.9%) who reported “slightly agree” or higher on SABAS items and were categorized as “high-risk users” Table 1 compares the demographic information, PSMU, IGD, mental well-being, and psychological distress across the three latent profiles. The “high-risk users” scored much worse in all mental problems and had higher PSMU and IGD risk than other groups, supporting the validity of the classification.

**Table 2:** Fit indices of the latent profile models

Classes	LogLike	AIC	BIC	SABIC	Entropy	BLRT_p
2	-611581	1223199	1223371	1223311	0.85	< 0.01
**3**	**-594669**	**1189391**	**1189626**	**1189543**	**0.82**	** < 0.01**
4	-589262	1178591	1178890	1178785	0.78	< 0.01
5	-586244	1172569	1172931	1172804	0.79	< 0.01
6	-583354	1166801	1167227	1167078	0.79	1

*AIC* Akaike information criterion, *BIC* Bayesian information criterion, *BLRT* bootstrap likelihood ratio test, *p* < 0.05 suggesting significant better performance

### ROC analysis

The classification labelled "high-risk users" as "PSU cases" and all others ("low-risk users" and "middle-risk users") as "non-cases". This classification served as the reference standard for the ROC analysis. The ROC curve (Figure S2) demonstrated a substantial AUC value (0.997, 95% CI, 0.996–0.997) for SABAS scores as predictors. Table 3 outlines the sensitivity, specificity, PPV, NPV, accuracy, and Youden index for potential SABAS cutoff points. The maximum Youden index corresponded to a SABAS cut-off point of 23 (Youden index = 0.949). This cut-off yielded a high sensitivity of 98.1%, specificity of 96.8%, PPV of 87.9%, NPV of 99.5%, and diagnostic accuracy of 97.1%. Though cut-off points of 24 or 25 also offered high accuracy, their sensitivity was comparatively low (0.89 for a cut-off of 24 and 0.71 for 25), which is a crucial factor for a screening tool. Therefore, the cut-off point of 23, which delivered a balance between sensitivity, specificity, and accuracy, was determined to be the most optimal.

**Table 3:** Cutoff points for SABAS

Cutoff points	Sensitivity	Specificity	Youden	PPV	NPV	Accuracy
20	1.00	0.80	1.80	0.54	1.00	0.826
21	1.00	0.86	1.86	0.63	1.00	0.873
22	1.00	0.92	1.92	0.74	1.00	0.921
**23**	**0.98**	**0.97**	**1.95**	**0.88**	**1.00**	**0.961**
24	0.89	0.99	1.88	0.97	0.97	0.980
25	0.71	1.00	1.71	1.00	0.94	0.958
26	0.56	1.00	1.56	1.00	0.91	0.931
27	0.44	1.00	1.44	1.00	0.88	0.908

*SABAS* Smartphone-Application Based Addiction Scale, *PPV* Positive predictive value, *NPV* Negative predictive value

Using a cutoff point of 23, the prevalence of PSU in our sample was 21.1% (*n* = 13,317.). Intergroup differences between participants with and without PSU were examined and presented in Table 4. No significant sex difference was observed between the two groups. However, participants with PSU were found to be older and more likely to be only children, junior high school students, and current smokers and drinkers (all *p* < 0.001). Adolescents presenting PSU demonstrated significantly elevated problematic technology use, compromised mental health, and severe psychological symptoms. This was evident by their higher scores on the IGDS9-SF, BSAMS, PHQ-9, GAD-7, SDQ, and PSQI scales, alongside lower scores on the WEMWBS and CDRISC scales. A large effect size for PTU and psychological problems was observed (Cohen's d > 0.8 for IGDS9-SF, BSAMS, PHQ-9, GAD-7, SDQ, and PSQI), suggesting a considerable association between PSU and these psychological outcomes. Moderate effect size was detected in mental well-being measures (Cohen’s d > 0.6 for WEMWBS and CDRISC). Critically, the presence of PSU was correlated with significantly higher risk for depression (OR, 5.18, 95%CI, 4.97–5.40), anxiety (OR, 5.18, 95%CI, 4.94–5.43), sleep disturbance (OR, 3.86, 95%CI, 3.70–4.01), IGD (OR, 27.8, 95%CI, 24.2–32.0), and PSMU (OR, 22.2, 95%CI, 19.0–25.9).

**Table 4:** Sample characteristics of PSU cases and non-cases

**Variable**	**Non-PSU**, *N* = 49,888^a^	**PSU**, *N* = 13,317^a^	***p*-value**^b^	**Cohen’s d/Odds ratio, 95%CI**
**Gender, female**	25,303 (51%)	6,704 (50%)	0.4	0.99 (0.95, 1.02)
**Age**	14.30 (1.67)	14.44 (1.55)	< 0.001	0.08
**School type**			< 0.001	1.12 (1.08.1.17)
Junior high school	15,386 (31%)	4,446 (33%)		
Senior high school	34,502 (69%)	8,871 (67%)		
**Current smoker, yes**	505 (1.0%)	381 (2.9%)	< 0.001	2.88 (2.52, 3.29)
**Current drinker, yes**	4,058 (8.1%)	2,660 (20%)	< 0.001	2.82 (2.67, 2.97)
**Living in urban, yes**	36,929 (74%)	9,942 (75%)	0.14	0.97 (0.93, 1.01)
**Only children, yes**	10,786 (22%)	3,118 (23%)	< 0.001	1.02 (1.01, 1.03)
**Left-behind children, yes**	17,493 (35%)	4,709 (35%)	0.5	0.99 (0.95, 1.02)
**SDQ-Emotional Symptoms**	2.66 (2.30)	4.56 (2.60)	< 0.001	0.806
**SDQ-Conduct Problems**	2.03 (1.45)	3.03 (1.71)	< 0.001	0.661
**SDQ-Hyperactivity Inattention**	3.34 (2.06)	5.19 (2.12)	< 0.001	0.889
**SDQ-Peer problems**	3.09 (1.54)	3.41 (1.70)	< 0.001	0.201
**SDQ-Prosocial behavior**	7.42 (2.07)	6.58 (2.12)	< 0.001	0.404
**SDQ total difficulties**	11.1 (5.3)	16.2 (5.7)	< 0.001	0.944
**PHQ9**	5.4 (4.5)	10.3 (5.8)	< 0.001	1.023
**WEMWBS**	48 (13)	40 (12)	< 0.001	0.648
**IGDS9SF**	13.6 (5.0)	21.6 (8.3)	< 0.001	1.362
**BSMAS**	9.9 (3.9)	15.1 (5.8)	< 0.001	1.174
**SABAS**	14 (5)	26 (3)	< 0.001	2.660
**CDRISC**	24 (9)	18 (8)	< 0.001	0.605
**GAD-7**	4.5 (3.8)	8.3 (4.8)	< 0.001	0.971
**PSQI**	3.7 (2.9)	6.3 (3.4)	< 0.001	0.862
**IGD, yes (IGDS9-SF ≥ 32)**	239 (0.5%)	1,574 (12%)	< 0.001	27.8 (24.2, 32.0)
**PSMU, yes (BSAMS ≥ 24)**	197 (0.4%)	1,077 (8.1%)	< 0.001	22.2 (19.0, 25.9)
**Sleep disturbance, yes (PSQI ≥ 6)**	11,507 (23%)	7,140 (56%)	< 0.001	3.86 (3.70, 4.01)
**Depression, yes (PHQ-9 ≥ 10)**	7,951 (16%)	6,599 (50%)	< 0.001	5.18 (4.97, 5.40)
**Anxiety, yes (GAD-7 ≥ 10)**	4,380 (8.8%)	4,433 (33%)	< 0.001	5.18 (4.94, 5.43)

*SDQ* Strength and Difficulties questionnaire, *PHQ9* 9-item Patient health questionnaire, *WEMWBS* Warwick-Edinburgh Mental Well-being Scale, *IGDS9-SF* 9-item Internet Gaming Disorder scale Short Form, *BSMAS* Bergen Social Media Addiction Scale, *SABAS* Smartphone-Application Based Addiction Scale, *GAD-7* 7-item Generalized Anxiety Disorder Scale (GAD-7) for anxiety, *PSQI* Pittsburgh sleep quality index, *CD-RISC* Connor-Davidson Resilience Scale, *PSU* problematic smartphone use, *IGD* internet gaming disorder, *PSMU* problematic social media use

^a^n (%); Mean (SD)

^b^Pearson’s Chi-squared test; t test

### The overlap of PSU, PSMU, and IGD

**Fig. 2:**
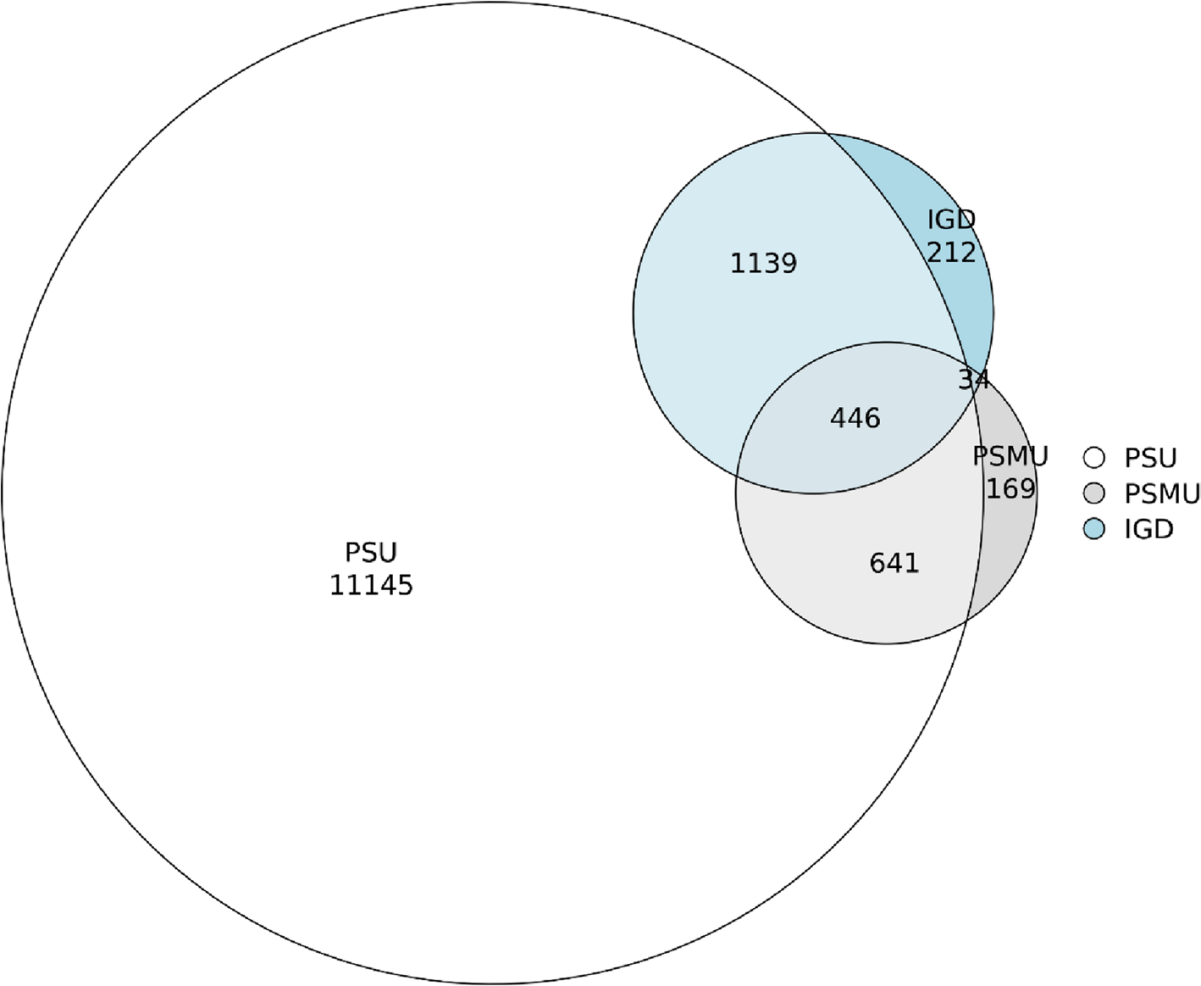
The overlap of PSMU, PSU, and IGD The Venn plot was used to visualize the overlap in the positive cases of PSMU, PSU, and IGD. Note: PSU: problematic smartphone use; IGD: internet gaming disorder; PSMU: problematic social media use

There was significant overlap among PSU, PSMU, and IGD cases. Specifically, 84.5% (1077 out of 1274) of the PSMU cases and 86.8% (1574 out of 1813) of the IGD cases concurrently fulfilled the criteria for PSU. Figure 2 illustrated this overlap. Of the participants, 21.7% (*n* = 13,721.) reported at least one subtype of PTU (PSU, PSMU, and IGD). A further breakdown revealed that 18.2% (*n* = 11,476.), 2.9% (*n* = 1,807), and 0.7% (*n* = 438) of participants experienced one, two, and three types of PTU, respectively.

Furthermore, for detecting IGD, PSU exhibited a sensitivity of 86.8%, specificity of 80.9%, PPV of 11.8%, NPV of 99.5%, and overall accuracy of 81.0%. In the context of identifying PSMU, the sensitivity was 84.5%, specificity 80.2%, PPV 8.1%, NPV 99.6%, and overall accuracy stood at 80.3%.

## Discussion

In this large-scale study of 63, 205. Chinese adolescents, we determined the optimal cutoff point for SABAS using the LPA and ROC approach. We also evaluated the ability of SABAS to capture IGD and PSMU. The major findings included: (1) LPA revealed a 3-class model of PSU, including low-risk users (38.6%), middle-risk users (42.5%), and high-risk users (18.9%). (2) ROC analysis demonstrated that 23 could be the optimal cutoff point for SABAS to detect PSU (sensitivity: 98.1%, specificity: 96.8%). Based on the cutoff point, 21% were identified as having PSU; (3) Adolescents with PSU exhibited much worse mental health and higher levels of PSMU and IGD; and (4) PSU screening with SABAS demonstrated satisfactory ability to capture IGD (sensitivity: 86.8%, specificity: 84.5%) and PSMU (sensitivity: 84.5%. specificity: 80.2%).

According to the responses to SABAS, our study demonstrated a three-class classification of PSU. The scoring pattern of the three classes for each SABAS item showed consistency, indicating that the classes differed in their item scores but did not show item preference. Furthermore, a strong positive association between PSU risk and mental distress was observed. Our findings were in line with studies in Iranian adolescents which also utilized SABAS to identify PSU [17]. In studies utilizing other measurements, there was no consensus on the optimal classification for smartphone users. Several studies have captured a similar three-class model [46–48], while other studies also proposed a four-class model [49, 50]. The different sample characteristics and contents of the utilized tool could contribute to the differences. Nevertheless, despite the different classifications and measurement tools, studies consistently report higher levels of mental distress in the class associated with the highest risk of PSU [48], which supported our present findings.

Combing LPA and ROC analysis, the study revealed an optimal cutoff point of 23 for SABAS to detect PSU, which achieved very high accuracy. Previous studies have utilized an empirical cutoff point of 21 in identifying PSU [23, 24]. However, this cutoff point yielded a relatively low PPV of 63%, suggesting that using such a cutoff point could result in a high number of false positives and potentially overestimation of the PSU prevalence.

Based on the derived cutoff point of 23, the incidence of PSU in our samples was 21%, which was fairly close to the findings reported by the meta-analysis in adolescents (21.6%) [6]. In line with prior reports [7, 51], PSU adolescents displayed much worse mental health. Notably, our study identified a significant overlap between PSU, PSMU, and IGD, positioning PSU as a broad, generalized condition encompassing PSMU and IGD. The majority of IGD and PSMU cases also fulfilled PSU criteria, aligning with prior findings of frequent coexistence of high levels of PSMU, IGD, and PSU [52]. These results resonate with the cognitive-behavioral model's division of PTU into generalized and specific conditions [34]. Further research is required to discern the factors contributing to the development of specific PTUs within the context of PSU.

Our study has several significant implications for both clinical practice and future research. First, it pioneers the establishment of a cut-off point for SABAS, enhancing the tool's utility in epidemiological research and interventions. Future studies can employ SABAS and its cut-off point to examine the prevalence of PSU and to identify adolescents needing further intervention for PSU. Second, our findings highlight PSU as the most prevalent form of PTU, affecting one in five adolescents, and being associated with substantial mental health problems. The high prevalence of PSU and its notable association with mental distress underscore the importance of regular PSU screenings among adolescents. Third, our results show a high sensitivity of PSU to detect IGD (86.8%) and PSMU (84.5%), suggesting that PTU screening using SABAS could be an effective method to detect specific PTU. Collectively, our study proposes a two-step screening process: initial PSU screening using SABAS, followed by assessments for specific PTU and mental distress.

There are several limitations to our study. First, the cross-sectional nature of our research prevents us from drawing causal inferences. Second, while the sample size was substantial, it was derived from a community sample of adolescents in a single city in China, restricting the immediate generalizability of our findings to other populations such as adults, adolescents from different cultural backgrounds, or clinical samples. Further research is required to validate these findings in more diverse settings. Third, SABAS was designed based on the six addiction components. Several important characteristics of PSU, such as daily life disturbance, were not measured. Forth, all questionnaires were self-reported, which might induce memory bias and social desirability bias. More importantly, LPA is exploratory. The identified cut-off point should be corroborated with the gold standards of clinical interviews and diagnosis in future research. In sum, these limitations suggest the need for further longitudinal research employing comprehensive assessments and clinical interviews.

## Conclusion

In summary, our study revealed that a cutoff point of 23 on SABAS can serve as an effective threshold for screening PSU in Chinese adolescents. 21% of the adolescents suffered from PSU. The considerable overlap observed between PSU, IGD, and PSMU, along with the significant association between PSU and mental distress, provides support for a promising two-step screening approach to identify PTU. SABAS could be administrated as an initial screening tool to detect PSU, followed by targeted assessments for specific PTU and mental health evaluations among individuals who screen positive.

### Supplementary Information

**Additional file 1: Figure S1.** The scree plot of AIC, BIC, SA-BIC of the 2-6 class solutions of the latent profile analysis.

**Additional file 2: Figure S2.** The ROC curve of SABAS to detect the PSU cases identified by latent profile analysis.

## Data Availability

The datasets used and/or analyzed during the current study are available from the corresponding author on reasonable request.

## Abbreviations

PSU: Problematic smartphone use
PSMU: Problematic smartphone use
PTU: Problematic technology use
IGD: Internet gaming disorder
PHQ-9: 9-Item Patient Health Questionnaire
GAD-7: 7-Item Generalized Anxiety Disorder Questionnaire
PSQI: Pittsburgh Sleep Quality Index
SABAS: Smartphone application-based addiction scale
BSAMS: Bergen Social Media Addiction Scale
SDQ: Strength and difficulties questionnaire
CD-RISC-10: Connor-Davidson Resilience Scale
WEMWBS: Warwick-Edinburgh Mental Well-being Scale
IGDS9-SF: Nine-Item Internet Gaming Disorder Scale-Short Form
LPA: Latent profile analysis
BIC: Bayesian information criterion
AIC: Akaike’s information criterion
BLRT: Bootstrap likelihood ratio
ROC: Receiver operating characteristic curve
PPV: Positive predictive value
NPV: Negative predictive value
