# Supplementary material for: Determination of the cutoff point for Smartphone Application-Based Addiction Scale for adolescents: a latent profile analysis
Source: BMC Psychiatry. 2023 Sep 16;23:675. doi: 10.1186/s12888-023-05170-4 (PMC10504767; doi:10.1186/s12888-023-05170-4)

Supplementary material for “Determination of the cutoff point for Smartphone Application-Based Addiction Scale for adolescents: a latent profile analysis ”

**Figure S1 The scree plot of AIC, BIC, SA-BIC of the 2-6 class solutions of the latent profile analysis**

Figure S1. The scree plot of AIC, BIC, SA-BIC of the 2-6 class solutions of the latent profile analysis


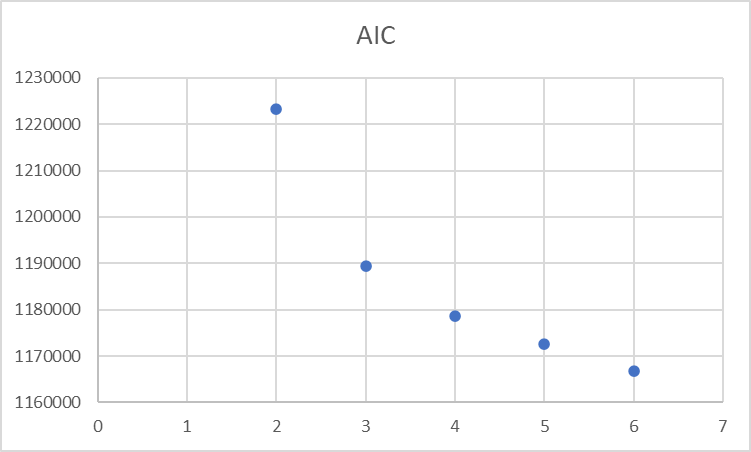


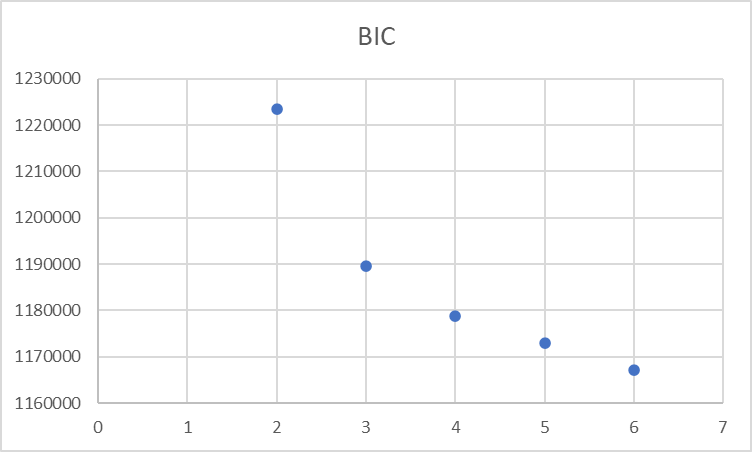


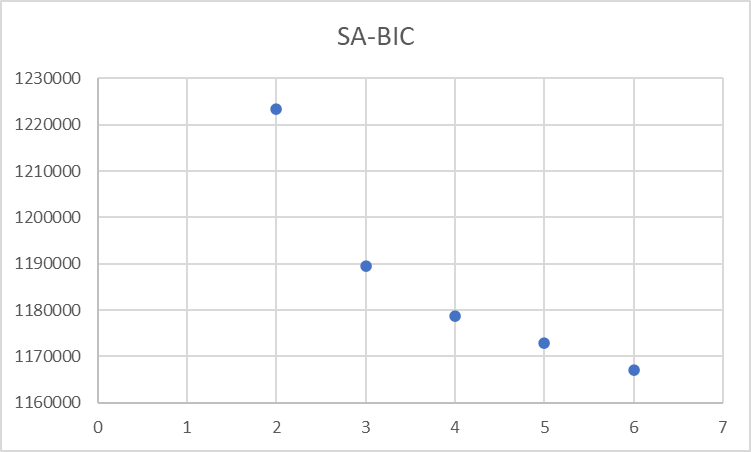

Supplement: Supplementary file 1 — Additional file 1: Figure S1. The scree plot of AIC, BIC, SA-BIC of the 2-6 class solutions of the latent profile analysis. [file 12888_2023_5170_MOESM1_ESM.docx]
