# Supplementary figures and images for: Determination of the cutoff point for Smartphone Application-Based Addiction Scale for adolescents: a latent profile analysis
Source: BMC Psychiatry. 2023 Sep 16;23:675. doi: 10.1186/s12888-023-05170-4 (PMC10504767; doi:10.1186/s12888-023-05170-4)

Figure S2 The ROC curve of SABAS to detect the PSU cases identified by latent profile analysis


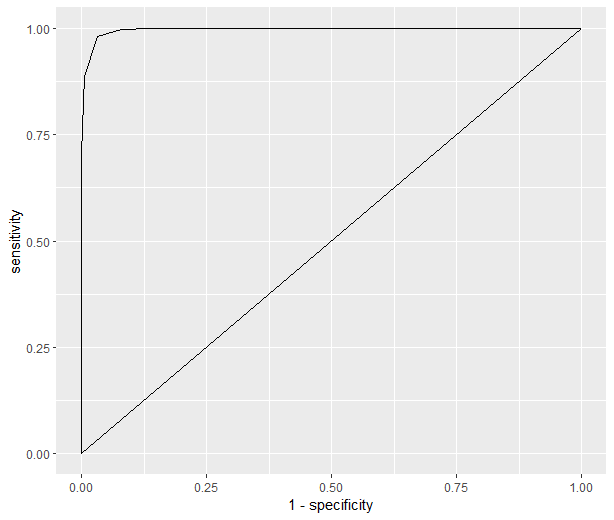

Supplement: Supplementary file 2 — Additional file 2: Figure S2. The ROC curve of SABAS to detect the PSU cases identified by latent profile analysis. [file 12888_2023_5170_MOESM2_ESM.docx]
